# The association of change in peak oxygen uptake with use of psychotropics in community-dwelling older adults - The Generation 100 study

**DOI:** 10.1186/s12877-022-03262-6

**Published:** 2022-07-13

**Authors:** Trude Carlsen, Dorthe Stensvold, Ulrik Wisløff, Linda Ernstsen, Thomas Halvorsen

**Affiliations:** 1 The Cardiac Exercise Research Group at Department of Circulation and Medical Imaging, Faculty of Medicine and Health Sciences, Norwegian University of Science and Technology, Trondheim, Norway; 2 Department of Public Health and Nursing, Faculty of Medicine and Health Sciences, Norwegian University of Science and Technology, Trondheim, Norway; 3 Department of Geography, Faculty of Social and Educational Sciences, Norwegian University of Science and Technology, Trondheim, Norway

**Keywords:** Aged, Cardiorespiratory fitness, Exercise, Antidepressants, Benzodiazepines, Z-hypnotics, Benzodiazepine-related medication

## Abstract

**Background:**

The use of psychotropics is high among the older population and may have detrimental effects on their physical and mental health. Cardiorespiratory fitness (CRF) is a strong and modifiable measure of health and declines with age. We aimed to study the association of change in CRF with use of psychotropics in community-dwelling older adults.

**Methods:**

We analyzed longitudinal data from 1531 older adults from the Generation 100 study, aged 70–77 years at inclusion, and with a permanent address in Trondheim, Norway. Data on objectively measured peak oxygen uptake (VO_2peak_) were linked with register data from the Norwegian Prescription Database on prescribed psychotropics. The included psychotropics were antidepressants (N06A), antipsychotics (N05A), anxiolytics (N05B), hypnotics and sedatives (N05C), and N03AE (benzodiazepine derivatives). Analyses were done on any psychotropics as one group, and on the following separate groups: antidepressants (N06A), benzodiazepines (N05BA, N05CD, and N03AE), and z-hypnotics (N05CF). Peak oxygen uptake was measured four times over a five-year period and corresponding medication use was measured as defined daily doses (DDD). A random effects estimator was applied to investigate the association of change in VO_2peak_ with the use of psychotropics.

**Results:**

We found a statistically significant curvilinear association of change in VO_2peak_ with use of any psychotropics and antidepressants. For VO_2peak_ up to ~ 40 ml/kg/min, each 1 ml/kg/min increase was associated by a 3.3 DDD and 2.5 DDD decrease in use of any psychotropics and antidepressants, respectively. A bottoming-out effect was found and increases in VO_2peak_ above ~ 40 ml/kg/min showed increased use of any psychotropics and antidepressants. However, the association of change in VO_2peak_ with use was stronger for changes in the lower continuum of VO_2peak_ levels and decreased with increasing VO_2peak_. No statistically significant association of change in VO_2peak_ with use of benzodiazepines and z-hypnotics were found. However, because of a non-randomized design, we cannot rule out the possibility of confounding by indication.

**Conclusions:**

The results of this study show a curvilinear association of change in VO_2peak_ with use of any psychotropics and antidepressants in older adults. This relationship adds a new viewpoint on the adverse effects of psychotropic use and should be considered in interventions and policies aimed at reducing psychotropic medication use among the older population.

**Supplementary Information:**

The online version contains supplementary material available at 10.1186/s12877-022-03262-6.

## Introduction

The use of prescription medication and polypharmacy for mental health disorders is increasing among the older population. Data from the United States showed an increase in use of psychotropic medication (hereby called psychotropics) from 8.5 to 12.5% over a five-year period [1]. In Australia, a 58.2% increase in dispensing of psychotropics was seen from 2000 to 2011, with antidepressants largely responsible for the increase [2]. An increase in prescription trends and costs are also seen in Europe [3–5].

The high use of psychotropics may have detrimental effects on older adults and it is reported that only about half of the older population who take psychotropics respond to these medications [6]. Psychotropics are associated with increased risk for fractures [7, 8]*,* falls [9]*,* cardiometabolic effects [10] and premature mortality [11, 12]. In addition, with increasing age comes a concomitant increase in older adults having physical and psychological conditions reducing quality of life and the ability to manage daily life activities. Ageing is characterized by reduced muscle mass and muscle strength [13], and kidney function [14]. Consequently, older adults may be more vulnerable to the side-effects of psychotropics due to changes in their pharmacokinetics and pharmacodynamics related to ageing [15, 16], and the harm may outweigh the benefit of taking them. Given the evidence of increased use of psychotropics with age, and older adults vulnerability to adverse side-effects it is important to consider actions which can reduce the use of psychotropics.

Cardiorespiratory fitness (CRF) is shown to be a strong predictor of overall health and reduces the risk of premature mortality [17]. Further, low levels of CRF are associated with increased risk of symptoms of mental disorders [18–21], indicating a connection between psychological and physical health. A meta-analysis found an increased risk of common mental health disorders in those with low and medium CRF compared to those with a high CRF, indicating a dose-response relationship between CRF and mental health disorders [22]. Previous research have shown that leisure-time physical activity is associated with decreased risk of use of any psychotropics [23, 24]. However, the relationship between CRF and use of psychotropic medication is not studied in the older population.

In the present study, we aimed to study the association of change in CRF with the use of any psychotropics in community-dwelling older adults. In addition, we studied the association of change in CRF with use of the individual psychotropics (1) antidepressants, (2) benzodiazepines, and (3) benzodiazepine-related medication (hereby called z-hypnotics), in the same population.

## Methods

All methods were performed in accordance with the relevant guidelines and regulations.

### Study population and data sources

**Fig. 1:**
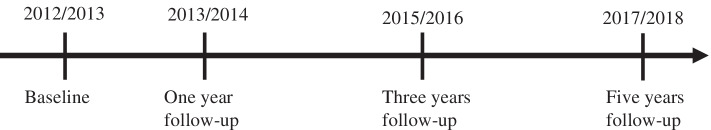
Timeline of the assessments in the study

The present study use data from the ongoing Generation 100 study; a large exercise intervention study conducted in Trondheim, Norway, with primary aim to determine the effects of long-term exercise with different intensities on mortality and morbidity in older adults [25, 26]. All women and men born between January 1, 1936, and December 31, 1942, with a permanent address in the municipality of Trondheim (*n* = 6966), received an invitation letter. A total of 1567 participants completed baseline testing and was included in the Generation 100 study, of whom 1531 participants provided data on the measures used in the present study. Participants were tested at four timepoints; baseline, one -, three -, and five years after inclusion (Fig. 1). A detailed description of the Generation 100 study protocol has previously been published [25].

We collected information about the use of prescribed psychotropics from the Norwegian Prescription Database (NorPD). The NorPD has since January 1, 2004, received monthly information from all pharmacies in Norway on all prescriptions dispensed to individuals outside institutions [27].

### Cardiopulmonary exercise testing

Cardiorespiratory fitness was measured as peak oxygen uptake (VO_2peak_) using Cortex MetaMax II (Cortex Biophysik Gmbh, Leipzig, Germany) or the Oxycon Pro (Erich Jaeger, Hoechberg, Germany, 3.8% of tests). The cardiopulmonary exercise testing (CPET) was based on an individualized protocol and performed walking or running to voluntary exhaustion on a treadmill (PPS55 Med, Woodway GmbH, Germany). Participants not able to perform the test on the treadmill due to physical constraints performed the test on a cycle ergometer (Monark cycle ergometer; 3.4% of tests). A description of the CPET protocol and equipment is previously described in detail [28]. Briefly, after a period of warm-up and submaximal workload, load was increased gradually by 2% inclination or 1 km/h (or 10 watts every 30 sec if cycling) each time oxygen uptake stabilized and until exhaustion or maximal oxygen uptake (VO_2max_) was reached. Criteria for achieving VO_2max_ was respiratory exchange ratio of 1.05 or higher, participant continued until exhaustion, and oxygen uptake did not increase more than 2 ml/kg/min between the last two workloads (levelling off VO_2_ despite increase in workload). Not all participants met the criteria for VO_2max_ (65% of men and 56% of women at baseline) and VO_2peak_ is therefore used throughout this paper.

### Psychotropic medication

The Anatomical Therapeutic Chemical (ATC) classification system and defined daily doses (DDD) are used as measuring unit as recommended by the World Health Organization [29]. The data extracted for this study include all dispensed prescriptions of psychotropics in the period between January 2012 through June 2018 for ATC codes N06A (antidepressants), N05A (antipsychotics), N05B (anxiolytics), N05C (hypnotics and sedatives), and N03AE (benzodiazepine derivatives), dispensing month and year, and number of DDD per dispensed prescription. The amount of dispensed psychotropics was calculated as DDDs, representing the average daily maintenance dose for a medication used for its main indication in adults [30]. The amount of psychotropics was defined at each follow-up as the aggregated number of DDDs over a year (eight months prior to and four months after each examination).

### Other variables

Body mass index (kg/m^2^) was calculated as body mass (kg) divided by height (m) squared and presented as a continuous value.

### Statistics

The data consists of repeated measurements of the same individuals over time. To account for serial correlation, we analyzed the association of VO_2peak_ with psychotropics using a panel data estimator. We applied the random effects estimator to take advantage of both cross-sectional variation (between individuals) and serial variation (within the same individual over time) in the estimation. This makes it potentially a more efficient estimator than other estimators that don’t make use of both dimensions of variation [31]. The random effects estimator does not require a balanced panel. Missing observations or unequal number of testing points are accepted.

The dependent variables in our models are the aggregated number of DDDs over a year for different groups of psychotropics and all psychotropics as one group. A list of all included psychotropics is listed in Additional file (Additional table 1). The explanatory variables of our models consist of sex, age, BMI and the measure of VO_2peak_ and a squared expression of the same measure to allow for non-linear relationships between VO_2peak_ and psychotropics. A prediction model was fitted from each estimation model. Twenty participants (1.3%) were prescribed antipsychotics at baseline. No individual analyses were done on this medication group due to few users of antipsychotics.

We also tested if there were differences in the association of change in VO_2peak_ with psychotropics in women and men. No statistically significant difference was found between women and men (additional tables 2, 4, 6 and 8), and the main analyses were therefore done for the total sample. Also, because women use more psychotropics and have lower VO_2peak_ than men, we did sex specific analyses which are presented in additional Tables 3, 5, 7 and 9.

All statistical analyses were performed using Stata 16 and 17 (StataCorp LLC, College Station, Texas, USA) and *p*-values lower than 0.05 were considered statistically significant. Values presented are mean (standard deviation) or number (N, (%)).

## Results

Baseline characteristics are shown in Table 1. Age was 72.4 (2.0) years, and the population was balanced across sexes (50% men).

**Table 1:** Baseline characteristics of the Generation 100 population

Characteristic	
Number of participants	1567
Any psychotropic medication, n (%)	366 (23.4)
Antidepressants, n (%)	102 (6.5)
Benzodiazepines, n (%)	126 (8.0)
Z-hypnotics, n (%)	227 (14.5)
Antipsychotics, n (%)	20 (1.3)
VO_2peak_ (ml/kg/min), mean (SD)	28.7 (6.4)
Body mass index (kg/m^2^), mean (SD)	26.0 (3.6)

Abbreviations: VO_2peak_ peak oxygen uptake, *SD* Standard deviation

### Use of any psychotropics

From first to fourth follow-up the number of participants using any psychotropics increased from 366 (23.4%) to 426 (27.2%). Across the four follow-ups the DDD (mean (SD)) was 41.6 (149.1), 42.7 (147.6), 53.4 (171.9) and 53.6 (160.5) at first, second, third and fourth follow-up, respectively, for the total sample.

**Fig. 2:**
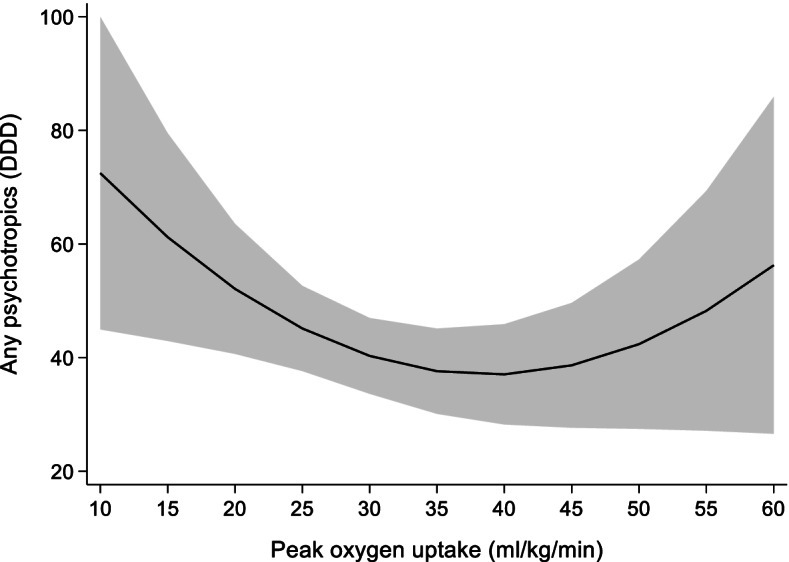
Prediction model for the association of change in VO_2peak_ with use of any psychotropics measured as DDD. DDD: defined daily doses, VO_2peak_: peak oxygen uptake. Shaded area = 95% confidence interval

The association between VO_2peak_ and the use of psychotropics are shown in Table 2. Each 1 ml/kg/min increase in VO_2peak_ shows a statistically significant association with reduction in use of psychotropics (*p* = 0.03). With increasing VO_2peak_ the association of change in VO_2peak_ with the use of any psychotropics is reduced (*p* = 0.04). The prediction model (Fig. 2) shows a curvilinear relationship between use of psychotropics and VO_2peak_. The highest psychotropics use is shown among the least fit with a VO_2peak_ of ~ 15 ml/kg/min using ~ 61 DDD of psychotropics. Psychotropics use decreases to ~ 37 DDD as VO_2peak_ increases to ~ 40 ml/kg/min. Increases in VO_2peak_ above ~ 40 ml/kg/min shows an increased use as a VO_2peak_ of ~ 58 ml/kg/min corresponds to a use of ~ 52 DDD.

**Table 2:** Random effects model showing the association of 1 ml/kg/min increase in VO_2peak_ with use of any psychotropics

	Coefficient	Standard error	95% confidence interval	
**VO**_**2peak**_	−3.3^*^	1.5	−6.3	− 0.4
**VO**_**2peak**_ **x VO**_**2peak**_	0.04^*^	0.02	0.001	0.08
**Sex**	41.3^**^	7.9	25.9	56.7
**Age**	1.0^*^	0.5	−0.002	2.0
**BMI**	1.5	1.1	−0.6	3.7
**Constant**	−31	55.2	− 139	77.2
**n**	4525			
**N**	1531			
**R**^**2**^	0.023			

Abbreviations: VO_2peak_ Peak oxygen uptake (ml/kg/min), *BMI* Body mass index (kg/m^2^), *n*: number of observations, N: number of unique individuals

Any psychotropics measured as defined daily doses

^**^ *p < 0.001,* ^*^ *p* < 0.05

### Antidepressants (ATC-CODE N06A)

From first to fourth follow-up the number of participants using antidepressants increased from 102 (6.5%) to 113 (7.2%). Across the four follow-ups the DDD (mean (SD)) was 14.1 (77.9), 13.5 (78), 19.6 (103.9) and 19.5 (104.5) at first, second, third and fourth follow-up, respectively, for the total sample.

**Fig. 3:**
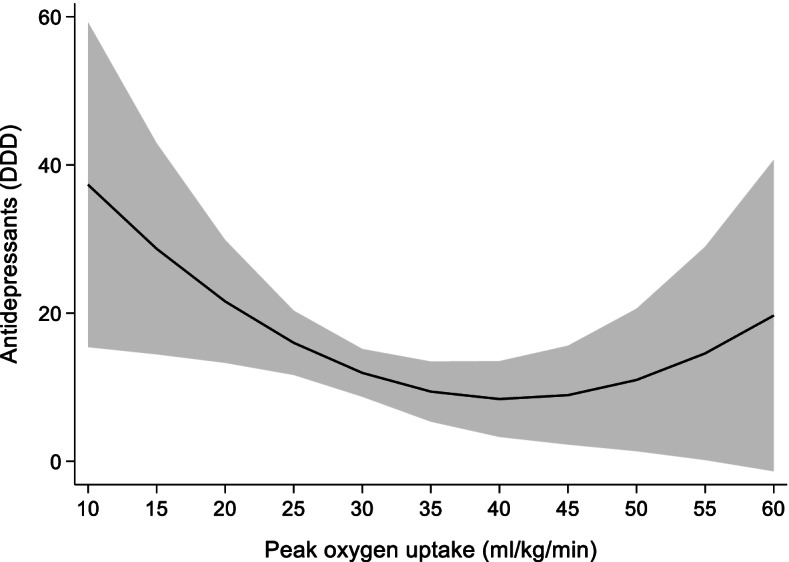
Prediction model for the association of change in VO_2peak_ with use of antidepressants measured as DDD. DDD: defined daily doses, VO_2peak_: peak oxygen uptake. Shaded area = 95% confidence interval

The association of 1 ml/kg/min change in VO_2peak_ with use of antidepressants are shown in Table 3. Each 1 ml/kg/min increase in VO_2peak_ is associated with a statistically significant reduction in use of antidepressants (*p* = 0.04). The prediction model (Fig. 3) shows a curvilinear relationship between use of antidepressants and VO_2peak_ with decreasing use as VO_2peak_ increases to ~ 40 ml/kg/min. A VO_2peak_ of ~ 15 ml/kg/min corresponds to a use of ~ 28 DDD and decreases to ~ 8.4 DDD with a VO_2peak_ of ~ 40 ml/kg/min. Antidepressant use increases to ~ 17 DDD at VO_2peak_ ~ 58 ml/kg/min.

**Table 3:** Random effects model showing the association of 1 ml/kg/min increase in VO_2peak_ with use of antidepressants

	Coefficient	Standard error	95% confidence interval	
**VO**_**2peak**_	−2.5^*^	1.2	−4.9	−0.1
**VO**_**2peak**_ **x VO**_**2peak**_	0.03	0.02	−0.002	0.06
**Sex**	11.4^*^	4.2	3.1	19.7
**Age**	0.03	0.36	−0.69	0.73
**BMI**	0.72	0.67	−0.6	2.0
**Constant**	57.8	33.5	−7.9	123.5
**n**	4525			
**N**	1531			
**R**^**2**^	0.011			

Abbreviations: VO_2peak_ Peak oxygen uptake (ml/kg/min), *BMI* Body mass index (kg/m^2^), n number of observations, *N* number of unique individuals

Antidepressants measured as defined daily doses

^*^ *p* < 0.05

### Benzodiazepines (ATC-CODES N05BA, N05CD AND N03AE)

From first to fourth follow-up the number of participants using benzodiazepines decreased from 126 (8%) to 120 (7.7%). Across the four follow-ups the DDD (mean (SD)) was 5.8 (36.3), 5.7 (34.9), 5.8 (34.7) and 5.2 (32.1) at first, second, third and fourth follow-up, respectively, for the total sample.

**Fig. 4:**
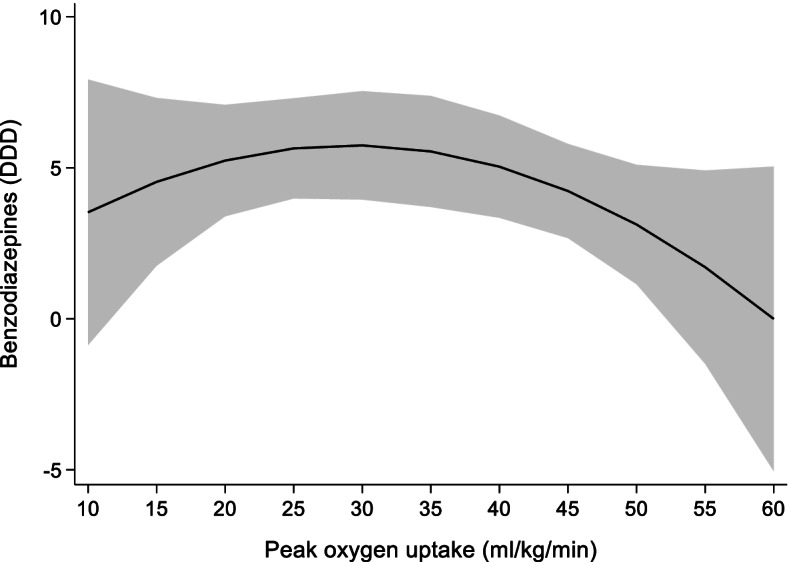
Prediction model for the association of change in VO_2peak_ on use of benzodiazepines measured as DDD. DDD: defined daily doses, VO_2peak_: peak oxygen uptake. Shaded area = 95% confidence interval.

The main results of the association of 1 ml/kg/min increase in VO_2peak_ with use of benzodiazepines are shown in Table 4. No statistically significant relationship was found between change in VO_2peak_ and use of benzodiazepines (*p* = 0.2, Fig. 4).

**Table 4:** Random effects model showing the association of 1 ml/kg/min increase in VO_2peak_ with use of benzodiazepines

	Coefficient	Standard error	95% confidence interval	
**VO**_**2peak**_	0.35	0.3	−0.2	0.9
**VO**_**2peak**_ **x VO**_**2peak**_	0.006	0.004	−0.01	0.002
**Sex**	4.8^*^	1.7	1.5	8.0
**Age**	−0.14	0.08	−0.3	0.03
**BMI**	−0.18	0.18	−0.54	0.18
**Constant**	13.3	10.3	−6.9	33.4
**n**	4525			
**N**	1531			
**R**^**2**^	0.005			

VO_2peak_ Peak oxygen uptake (ml/kg/min), *BMI* Body mass index (kg/m^2^), *n* number of observations, *N* number of unique individuals.

Benzodiazepines measured as defined daily doses

^*^ *p < 0.05*

### Z-hypnotics (N05CF)

From first to fourth follow-up the number of participants using z-hypnotics increased from 227 (14.5%) to 289 (18.4%). Across the four follow-ups the DDD (mean (SD)) was 16.2 (67.8), 19.2 (74.5), 23.6 (87) and 25.2 (85.2) at first, second, third and fourth follow-up, respectively, for the total sample.

**Fig. 5:**
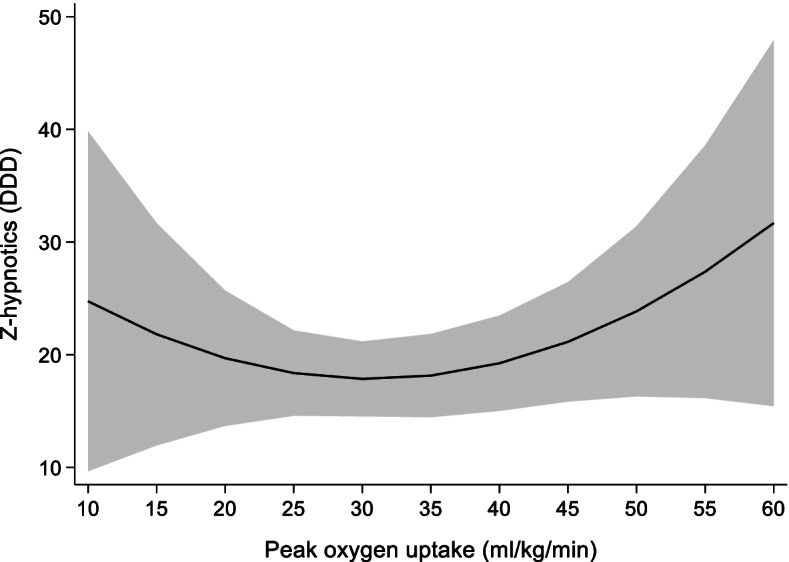
Prediction model for the association of change in VO_2peak_ with use of z-hypnotics measured as DDD. *DDD* defined daily doses, VO_2peak_ peak oxygen uptake. Shaded area = 95% confidence interval.

The main results of the association of 1 ml/kg/min increase in VO_2peak_ with use of Z-hypnotics are shown in Table 5. No statistically significant relationship was found between change in VO_2peak_ and use of z-hypnotics (*p* = 0.25, Fig. 5).

**Table 5:** Results from random effects model showing the association of 1 ml/kg/min increase in VO_2peak_ on use of z-hypnotics

	Coefficient	Standard error	95% confidence interval	
**VO**_**2peak**_	−1.0	0.86	−2.7	0.69
**VO**_**2peak**_ **x VO**_**2peak**_	0.02	0.01	−0.008	0.04
**Sex**	17.9^**^	3.7	10.6	25.2
**Age**	1.2^**^	0.26	0.7	1.71
**BMI**	0.26	0.51	−0.74	1.27
**Constant**	−72^*^	28.7	− 128	− 15.8
**n**	4525			
**N**	1531			
**R**^**2**^	0.01			

VO_2peak_ Peak oxygen uptake (ml/kg/min), *BM* Body mass index (kg/m^2^), *n* number of observations, *N* number of unique individuals.

Z-hypnotics measured as defined daily doses

^*^ *p < 0.05,* ^**^ *p < 0.001*

### Sex specific analyses

Our analyses show no statistically significant difference in the association of change in VO_2peak_ with psychotropics (any psychotropics and separate psychotropic groups) between women and men. Additional file shows this in further detail. However, the bottoming-out effect of the association of change in VO_2peak_ with psychotropics appeared at lower VO_2peak_ levels in women than men, ~ 30–35 ml/kg/min and ~ 40 ml/kg/min for women and men, respectively.

## Discussion

Our data indicate a curvilinear association of change in VO_2peak_ with use of any psychotropics in older adults. The same pattern was seen when studying antidepressants and z-hypnotics as separate medication groups. However, this pattern was only significant for antidepressants. Furthermore, we found a sex difference in use of all psychotropic medication groups with women using more psychotropics compared to men. We also tested the association of change in VO_2peak_ with use of psychotropics between sexes, but no statistically significant sex differences were found for any of the medication groups.

No studies have investigated the association of change in VO_2peak_ and prescribed psychotropics in older adults which make comparison with other studies challenging. Given the evidence of increasing use of psychotropics among the older population it is important to consider actions to reduce this trend. The present study adds to the literature on the relationship between physical and mental health in older adults, and how changes in physical health (VO_2peak_) is associated with use of psychotropics.

Previous studies have found an inverse relationship between CRF and risk of common mental health disorders, with greater risk among those with low and medium CRF compared to those with high CRF [22, 32, 33]. Our study shows that increasing VO_2peak_ is associated with a decrease in psychotropics use. Any decrease in use could have a significant impact, as harmful side-effects have been reported from the use of these medications. However, the curvilinear relationship we found might indicate a bottoming-out effect on a possible treatment effect of psychotropics by changing VO_2peak_. Our results suggest that the treatment potential is larger in less fit older adults than for fit older adults. In addition to maintain VO_2peak_ for general health benefits, other treatment options should be considered to reduce psychotropics use for older adults with high fitness levels.

Studies investigating the relationship between PA and psychotropics use have showed a reduced risk of purchasing psychotropics by increasing physical activity levels with the lowest risk among those showing PA pattern of moderate to vigorous intensity of high volume [23]. The latter finding indicate that intensity could be an important PA dimension for psychotropics reduction and is also shown to be a key factor to increase VO_2peak_ [34, 35]_._

Stubbs et al. [24] found that only those being persistently inactive remained at increased risk for psychotropics use after adjusting for potential confounders, indicating that change in physical activity levels could play an important role in psychotropics prescribing. Even if CRF is largely influenced by physical activity levels none of the studies looking at physical activity and psychotropics use included CRF as a measure.

Measure of CRF is shown to have an independent predictive value for physical health [17] but the impact on psychotropics is less studied. Given the vulnerability for side-effects and increased comorbidities in older adults, our results indicate that those on the lower end of the CRF continuum will have a lot to gain by increasing CRF as psychotropics use decreases with small increases in CRF. Furthermore, our u-shaped relationship between VO_2peak_ and any psychotropics in older adults might indicate that individuals on the higher end of the CRF continuum might benefit more from actions other than increasing CRF for the purpose of reducing use of psychotropics. However, it should be noted that few older adults in our study had a VO_2peak_ above 40 ml/kg/min which might influence the pattern of association in the higher continuum of VO_2peak_ levels. The latter is also reflected by wide confidence intervals.

In addition to any psychotropics we studied the separate groups of psychotropics: antidepressants, benzodiazepines and z-hypnotics. We found the same curvilinear association of change in VO_2peak_ and use of antidepressants as we did for psychotropics considered as one medication group. Previous studies report an inverse relationship between CRF and depression disorders, including depressive symptoms both in healthy [32, 33, 36, 37] and those diagnosed with depression [36]. Our study found no statistically significant association of change in VO_2peak_ with use of either benzodiazepines or z-hypnotics. Higher risk of anxiety is found among those with low and medium CRF compared with high CRF [22]. Two studies have previously investigated the association between insomnia symptoms and CRF, and both reported a modest inverse relationship [20, 21].

Our study contributes with new insights on the topic of psychotropic use among older adults. Strategies to increase older adults’ VO_2peak_ to optimal levels for reduced psychotropics use does not seem to follow “the more the better” principle. Also, in addition to promote mental health, improving VO_2peak_ can improve cardiovascular health and reduce all-cause mortality [38, 39]. A recent Lancet Commission paper highlights that focus should be on strategies which target both physical and mental health [40]. However, given the curvilinear relationship between change in VO_2peak_ and psychotropics use, VO_2peak_ should be measured and monitored to tailor interventions to the individual VO_2peak_ level.

### Study strengths and limitations

A strength in our study is complete national register data on psychotropic medication dispensing linked with direct and objectively measured VO_2peak_ data from a large sample of older adults. We have four measurement points of VO_2peak_ over five years. In addition, direct and objectively measured VO_2peak_ is considered the gold standard for measuring CRF.

A limitation of the study is that information on dispensed psychotropics is not a direct measure of mental health issues and comparison with studies reporting other measures than medication use is difficult. Data on dispensed psychotropics were only available eight months before first follow-up which means that the aggregated one-year period for all follow-ups are eight months before and four months after the follow-ups. However, register-based data gives only information about dispensed medication and not if or when they are taken. Further, we lack information on what specific cause these medications were prescribed for. Comorbidity exists between mental health disorders and polypharmacy is common in older adults. Also, psychotropics are not only related to mental health problems as some psychotropics are also prescribed for somatic conditions. Therefore, we chose to do the main analysis on any psychotropics which we think best indicate general mental health.

Concerning the separate psychotropics groups, antipsychotic medication is prescribed for severe mental disorders and the few users in the present study may indicate that this group is underrepresented in exercise interventions targeting the general older adult population. Importantly, the study population was recruited based on selected inclusion criteria to attend an exercise intervention and there are 28–63% less users of the different groups of psychotropics in the study population compared with age-matched peers in Norway [41]. The bottoming-out effect was seen when VO_2peak_ were ~ 40 ml/min/kg which is considered a high VO_2peak_ for this age group. The latter implies that many older adults will benefit from increasing their VO_2peak_.

The possibility of unmeasured confounding cannot be ruled out which may have influenced both the exposure (change in VO_2peak_) and the outcome (psychotropics use). Lastly, given that neither change in VO_2peak_ nor psychotropics use are randomized we cannot discount the possibility of confounding by indication.

## Conclusion

The results of this study show a curvilinear association of change in VO_2peak_ with use of any psychotropics and antidepressants in older adults. Increases in VO_2peak_ is associated with decreases in use of any psychotropics and antidepressants, measured as DDD, but a bottoming-out effect is present, and the use increases as VO_2peak_ reaches higher levels. This relationship adds new insight into psychotropics use in the older population and should be considered in the clinical strategies for reducing medication.

## Supplementary Information

**Additional file 1.** An additional file (Additional file.docx) is provided with the manuscript with tables presenting a list of included medication groups and the results of the analyses including the interaction term of VO_2peak_ and sex, and for women and men separately. These analyses where done for any psychotropics and the separate psychotropic groups.

## Data Availability

The data analyzed in the present study was obtained from the Generation 100 Study and Norwegian Prescription Database. Contact information on access to data from the Generation 100 Study are available at https://www.ntnu.edu/cerg/generation100. Data from the Generation 100 Study can be linked with data from the Norwegian Prescription Database. Information on how to apply can be found at https://helsedata.no/en/.

## Abbreviations

CRF: Cardiorespiratory fitness
DDD: Defined daily doses
NorPD: Norwegian Prescription Database
PA: Physical activity
VO_2max_: Maximal oxygen uptake
VO_2peak_: Peak oxygen uptake
