# Supplementary material for: The association of change in peak oxygen uptake with use of psychotropics in community-dwelling older adults - The Generation 100 study
Source: BMC Geriatr. 2022 Jul 13;22:575. doi: 10.1186/s12877-022-03262-6 (PMC9281052; doi:10.1186/s12877-022-03262-6)
Supplement: Supplementary file 1 — Additional file 1. An additional file (Additional file.docx) is provided with the manuscript with tables presenting a list of included medication groups and the results of the analyses including the interaction term of VO2peak and sex, and for women and men separately. These analyses where done for any psychotropics and the separate psychotropic groups. [file 12877_2022_3262_MOESM1_ESM.docx]

**Additional table 1.** List of medications used in the study.

| **Medication group:** | **Medication groups included according to ATC-classification:** |
| --- | --- |
| Antidepressants | **N06A**  **N06AA Non-selective monoamine reuptake inhibitors**  N06AA04 clomipramine  N06AA06  trimipramine  N06AA09 amitriptyline  N06AA10 nortriptyline  N06AA12 doxepin  **N06AB Selective serotonin reuptake inhibitors**  N06AB03 fluoxetine  N06AB04 citalopram  N06AB05 paroxetine  N06AB06 sertraline  N06AB10 escitalopram  **N06AG Monoamine oxidase A inhibitors**  N06AG02 moclobemide  **N06AX Other antidepressants**  N06AX03 mianserin  N06AX11 mirtazapine  N06AX12 bupropion  N06AX16 venlafaxine  N06AX21 duloxetine |
| Antipsychotics | **N05A**  **N05AA Phenothiazines with aliphatic side-chain**  N05AA02 levomepromazine  N05AB04 prochlorperazine  N05AD01 haloperidol  N05AF01 flupentixol  N05AF03 chlorprothixene  N05AH03 olanzapine  N05AH04 quetiapine  N05AN01 lithium  N05AX08 risperidone |
| Anxiolytics | **N05B**  **N05BA Benzodiazepine derivatives**  N05BA01 diazepam  N05BA04 oxazepam  **N05BB Diphenylmethane derivatives**  N05BB01 hydroxyzine  **N05BE Azaspirodecanedione derivatives**  N05BE01 buspirone |
| Hypnotics and sedatives | **N05C**  **N05CD Benzodiazepine derivatives**  N05CD02 nitrazepam  N05CD03 flunitrazepam  N05CD08 midazolam  **N05CF Benzodiazepine related drugs**  N05CF01 zopiclone  N05CF02 zolpidem  **N05CH Melatonin receptor agonists**  N05CH01 melatonin |
| Benzodiazepines | **N05BA Benzodiazepine derivatives**  N05BA01 diazepam  N05BA04 oxazepam  **N05CD Benzodiazepine derivatives**  N05CD02 nitrazepam  N05CD03 flunitrazepam  N05CD08 midazolam  **N03AE Benzodiazepine derivatives**  N03AE01 clonazepam |
| Z-hypnotics | **N05CF Benzodiazepine related drugs**  N05CF01 zopiclone  N05CF02 zolpidem |

**Additional analyses**

**Any psychotropics**

**Additional table 2**. Random effects model showing the association of 1 ml/kg/min
increase in VO_2peak_ with use of any psychotropics in the total sample. Interaction with sex.

|  | Coefficient | Standard error | 95% confidence interval | |
| --- | --- | --- | --- | --- |
| **VO_2peak_** | -3.0 | 1.9 | -6.8 | 0.73 |
| **VO_2peak_ x VO_2peak_** | 0.04 | 0.03 | -0.01 | 0.09 |
| **Sex** | 61.8 | 58 | -51.4 | 175 |
| **Sex x VO_2peak_** | -1.75 | 3.5 | -8.6 | 5.1 |
| **Age** | 1.0 | 0.51 | -0.01 | 2.0 |
| **BMI** | 1.5 | 1.1 | -0.63 | 3.6 |
| **Constant** | -33.1 | 62.3 | -155 | 89 |
| **n** | 4525 |  |  |  |
| **N** | 1531 |  |  |  |
| **R^2^** | 0.02 |  |  |  |

VO_2peak_: Peak oxygen uptake (ml/kg/min), BMI: Body mass index (kg/m^2^), n: number of
observations, N: number of unique individuals.
Any psychotropics measured as defined daily doses.

**Additional table 3**. Random effects model showing the association of 1 ml/kg/min
increase in VO_2peak_ with use of any psychotropics in women and men.

|  | Women | | | | Men | | | |
| --- | --- | --- | --- | --- | --- | --- | --- | --- |
|  | Coefficient | Standard error | 95% confidence interval | | Coefficient | Standard error | 95% confidence interval | |
| **VO_2peak_** | -4.4 | 2.9 | -10.1 | 1.27 | -3.8 | 2.0 | -7.6 | 0.05 |
| **VO_2peak_ x VO_2peak_** | 0.07 | 0.05 | -0.03 | 0.16 | 0.04 | 0.03 | -0.007 | 0.1 |
| **Age** | 0.96 | 0.84 | -0.69 | 2.6 | 0.79 | 0.57 | -0.33 | 1.92 |
| **BMI** | 2.71 | 1.74 | -0.7 | 6.1 | 0.19 | 1.15 | -2.1 | 2.4 |
| **Constant** | -8.6 | 84.4 | -174 | 157 | 30.2 | 70.6 | -108 | 169 |
| **n** | 2239 |  |  |  | 2286 |  |  |  |
| **N** | 769 |  |  |  | 762 |  |  |  |
| **R^2^** | 0.003 |  |  |  | 0.01 |  |  |  |

VO_2peak_: Peak oxygen uptake (ml/kg/min), BMI: Body mass index (kg/m^2^), n: number of observations, N: number of unique
individuals.
Any psychotropics measured as defined daily doses.

**Antidepressants**

**Additional table 4**. Random effects model showing the association of 1 ml/kg/min
increase in VO_2peak_ with use of antidepressants in the total sample. Interaction with sex.

|  | Coefficient | Standard error | 95% confidence interval | |
| --- | --- | --- | --- | --- |
| **VO_2peak_** | -2.8 | 1.6 | -6.0 | 0.44 |
| **VO_2peak_ x VO_2peak_** | 0.03 | 0.02 | -0.01 | 0.08 |
| **Sex** | 9.5 | 47.0 | -82.5 | 101 |
| **Sex x VO_2peak_** | -0.25 | 2.8 | -5.8 | 5.3 |
| **Age** | 0.03 | 0.36 | -0.67 | 0.72 |
| **BMI** | 0.75 | 0.67 | -0.56 | 2.06 |
| **Constant** | 38.4 | 42.2 | -44.4 | 121 |
| **n** | 4525 |  |  |  |
| **N** | 1531 |  |  |  |
| **R^2^** | 0.01 |  |  |  |

VO_2peak_: Peak oxygen uptake (ml/kg/min), BMI: Body mass index (kg/m^2^), n: number of
observations, N: number of unique individuals.
Antidepressants measured as defined daily doses.

**Additional table 5**. Random effects model showing the association of 1 ml/kg/min increase in VO_2peak_ with use of antidepressants in women and men.

|  | Women | | | | Men | | | |
| --- | --- | --- | --- | --- | --- | --- | --- | --- |
|  | Coefficient | Standard error | 95% confidence interval | | Coefficient | Standard error | 95% confidence interval | |
| **VO_2peak_** | -2.96 | 2.3 | -7.4 | 1.5 | -2.8 | 1.6 | -6.0 | 0.45 |
| **VO_2peak_ x VO_2peak_** | 0.04 | 0.04 | -0.03 | 0.12 | 0.03 | 0.02 | -0.01 | 0.08 |
| **Age** | -0.17 | 0.6 | -1.4 | 1.03 | 0.22 | 0.37 | -0.5 | 0.95 |
| **BMI** | 1.1 | 1.02 | -0.9 | 3.1 | 0.34 | 0.8 | -1.23 | 1.92 |
| **Constant** | 52.4 | 56.7 | -58.8 | 163 | 35.2 | 42.0 | -47.2 | 117.5 |
| **n** | 2239 |  |  |  | 2286 |  |  |  |
| **N** | 769 |  |  |  | 762 |  |  |  |
| **R^2^** | 0.003 |  |  |  | 0.01 |  |  |  |

VO_2peak_: Peak oxygen uptake (ml/kg/min), BMI: Body mass index (kg/m^2^), n: number of observations, N: number of unique individuals. Antidepressants measured as defined daily doses.

**Benzodiazepines**

**Additional table 6**. Random effects model showing the association of 1 ml/kg/min
increase in VO_2peak_ with use of benzodiazepines in the total sample. Interaction with sex.

|  | Coefficient | Standard error | 95% confidence interval | |
| --- | --- | --- | --- | --- |
| **VO_2peak_** | 0.43 | 0.46 | -0.48 | 1.33 |
| **VO_2peak_ x VO_2peak_** | -0.007 | 0.007 | -0.02 | 0.006 |
| **Sex** | 6.2 | 9.5 | -12.4 | 24.8 |
| **Sex x VO_2peak_** | -0.03 | 0.61 | -1.23 | 1.18 |
| **Age** | -0.14 | 0.08 | -0.30 | 0.03 |
| **BMI** | -0.19 | 0.18 | -0.55 | 0.17 |
| **Constant** | 11.8 | 11.0 | -9.8 | 33.4 |
| **n** | 4525 |  |  |  |
| **N** | 1531 |  |  |  |
| **R^2^** | 0.005 |  |  |  |

VO_2peak_: Peak oxygen uptake (ml/kg/min), BMI: Body mass index (kg/m^2^), n: number of
observations, N: number of unique individuals.
Benzodiazepines measured as defined daily doses.

**Additional table 7**. Random effects model showing the association of 1 ml/kg/min increase in VO_2peak_ with use of benzodiazepines in women and men.

|  | Women | | | | Men | | | |
| --- | --- | --- | --- | --- | --- | --- | --- | --- |
|  | Coefficient | Standard error | 95% confidence interval | | Coefficient | Standard error | 95% confidence interval | |
| **VO_2peak_** | 0.54 | 0.40 | -0.24 | 1.32 | 0.14 | 0.31 | -0.46 | 0.74 |
| **VO_2peak_ x VO_2peak_** | -0.009 | 0.006 | -0.02 | 0.003 | -0.003 | 0.005 | -0.01 | 0.006 |
| **Age** | -0.14 | 0.11 | -0.35 | 0.08 | -0.15 | 0.14 | -0.42 | 0.11 |
| **BMI** | -0.02 | 0.28 | -0.57 | 0.52 | -0.22 | 0.18 | -0.58 | 0.14 |
| **Constant** | 11.0 | 14.2 | -16.8 | 38.7 | 18.9 | 14.0 | -8.5 | 46.3 |
| **n** | 2239 |  |  |  | 2286 |  |  |  |
| **N** | 769 |  |  |  | 762 |  |  |  |
| **R^2^** | 0.0001 |  |  |  | 0.002 |  |  |  |

VO_2peak_: Peak oxygen uptake (ml/kg/min), BMI: Body mass index (kg/m^2^), n: number of observations, N: number of unique individuals. Benzodiazepines measured as defined daily doses.

**Z-hypnotics**

**Additional table 8**. Random effects model showing the association of 1 ml/kg/min
increase in VO_2peak_ with use of z-hypnotics in the total sample. Interaction with sex.

|  | Coefficient | Standard error | 95% confidence interval | |
| --- | --- | --- | --- | --- |
| **VO_2peak_** | -0.81 | 0.93 | -2.6 | 1.0 |
| **VO_2peak_ x VO_2peak_** | 0.01 | 0.01 | -0.01 | 0.04 |
| **Sex** | 27.7 | 32.4 | -35.8 | 91.1 |
| **Sex x VO_2peak_** | -0.76 | 2.0 | -4.6 | 3.1 |
| **Age** | 1.2 | 0.26 | 0.7 | 1.71 |
| **BMI** | 0.26 | 0.51 | -0.75 | 1.26 |
| **Constant** | -74 | 29.9 | -132 | -15.4 |
| **n** | 4525 |  |  |  |
| **N** | 1531 |  |  |  |
| **R^2^** | 0.01 |  |  |  |

VO_2peak_: Peak oxygen uptake (ml/kg/min), BMI: Body mass index (kg/m^2^), n: number of
observations, N: number of unique individuals.
Z-hypnotics measured as defined daily doses.

**Additional table 9**. Random effects model showing the association of 1 ml/kg/min increase in VO_2peak_ with use of z-hypnotics in women and men.

|  | Women | | | | Men | | | |
| --- | --- | --- | --- | --- | --- | --- | --- | --- |
|  | Coefficient | Standard error | 95% confidence interval | | Coefficient | Standard error | 95% confidence interval | |
| **VO_2peak_** | -1.33 | 1.73 | -4.7 | 2.1 | -1.12 | 0.96 | -3.0 | 0.75 |
| **VO_2peak_ x VO_2peak_** | 0.03 | 0.03 | -0.03 | 0.08 | 0.02 | 0.01 | -0.01 | 0.04 |
| **Age** | 1.47 | 0.39 | 0.71 | 2.23 | 0.83 | 0.34 | 0.17 | 1.5 |
| **BMI** | 0.46 | 0.79 | -1.1 | 2.0 | 0.05 | 0.64 | -1.2 | 1.3 |
| **Constant** | -63.5 | 40.2 | -142 | 15.3 | -34.4 | 40.1 | -113 | 44.1 |
| **n** | 2239 |  |  |  | 2286 |  |  |  |
| **N** | 769 |  |  |  | 762 |  |  |  |
| **R^2^** | 0.001 |  |  |  | 0.0002 |  |  |  |

VO_2peak_: Peak oxygen uptake (ml/kg/min), BMI: Body mass index (kg/m^2^), n: number of observations, N: number of unique individuals. Z-hypnotics measured as defined daily doses.
